# Feasibility assessment of invigorating grassrooTs primary healthcare for prevention and management of cardiometabolic diseases in resource-limited settings in China, Kenya, Nepal, Vietnam (the FAITH study): rationale and design

**DOI:** 10.1186/s41256-019-0124-0

**Published:** 2019-11-12

**Authors:** Enying Gong, Hongsheng Lu, Shuai Shao, Xuanchen Tao, Nicholas Peoples, Brandon A. Kohrt, Shangzhi Xiong, Catherine Kyobutungi, Tilahun Nigatu Haregu, Christopher Khayeka-Wandabwa, Hoang Van Minh, Tran Thi Duc Hanh, Suraj Koirala, Kamal Gautam, Lijing L. Yan

**Affiliations:** 1 Global Health Research Center, Duke Kunshan University, No. 8 Duke Avenue, Kunshan, 215316 Jiangsu China; 2 Global Health Institute, Duke University, Durham, NC USA; 3 Department of Psychiatry, George Washington University, Washington, DC USA; 4 African Population and Health Research Center, Nairobi, Kenya; 5 Hanoi University of Public Health, Hanoi, Vietnam; 6 Transcultural Psychosocial Organization Nepal, Kathmandu, Nepal

**Keywords:** Cardiometabolic diseases, Primary health care, Developing countries, Resource-limited settings

## Abstract

**Background:**

Cardiometabolic diseases are the leading cause of death and disability in many low- and middle-income countries. As the already severe burden from these conditions continues to increase in low- and middle-income countries, cardiometabolic diseases introduce new and salient public health challenges to primary health care systems. In this mixed-method study, we aim to assess the capacity of grassroots primary health care facilities to deliver essential services for the prevention and control of cardiometabolic diseases. Built on this information, our goal is to propose evidence-based recommendations to promote a stronger primary health care system in resource-limited settings.

**Methods:**

The study will be conducted in resource-limited settings in China, Kenya, Nepal, and Vietnam using a mixed-method approach that incorporates a literature review, surveys, and in-depth interviews. The literature, statistics, and document review will extract secondary data on the burden of cardiometabolic diseases in each country, the existing policies and interventions related to strengthening primary health care services, and improving care related to non-communicable disease prevention and control. We will also conduct primary data collection. In each country, ten grassroots primary health care facilities across representative urban-rural regions will be selected. Health care professionals and patients recruited from these facilities will be invited to participate in the facility assessment questionnaire and patients’ survey. Stakeholders – including patients, health care professionals, policymakers at the local, regional, and national levels, and local authorities – will be invited to participate in in-depth interviews. A standard protocol will be designed to allow for adaption and localization in data collection instruments and procedures within each country.

**Discussion:**

With a special focus on the capacity of primary health care facilities in resource-limited settings in low- and middle-income countries, this study has the potential to add new evidence for policymakers and academia by identifying the most common and significant barriers primary health care services face in managing and preventing cardiometabolic diseases. With these findings, we will generate evidence-based recommendations on potential strategies that are feasible for resource-limited settings in combating the increasing challenges of cardiometabolic diseases.

## Background

Cardiometabolic diseases (CMDs), including both cardiovascular diseases and diabetes mellitus, are the leading cause of death and disability across the globe [1]. According to new estimates from the Global Burden of Disease study, cardiovascular diseases and diabetes mellitus resulted in 17.79 million and 1.37 million global deaths, respectively, in 2017, which had increased by 21.1 and 34.8% since 2007 [1]. Although previous research on CMDs has largely focused on high-income countries, CMDs have become an increasing challenge to most low- and middle-income countries (LMICs) where about 80% of non-communicable disease (NCD)-related deaths occur. Exposure to risk factors for metabolic diseases has also increased in most LMICs in the past three decades [2]. CMDs further impose a substantial economic burden on LMICs: the anticipated economic burden of cardiovascular diseases in LMICs between 2011 and 2025 will be 3.76 trillion USD during the period of 2011 to 2025 [3]. This rapidly increasing burden of CMDs in LMICs needs immediate action in policies, health care services, and among the public.

Strengthening primary health care (PHC) is a key strategy for effective NCDs prevention and control [4–7]. Decades of evidence show that a strong PHC system rooted in the community can support the delivery of high-quality health care services at a lower cost than non-community based support and can respond to evolving health challenges [8, 9]. In 2010, the World Health Organization published its “Package of Essential NCD interventions for primary care in low-resource settings” (WHO PEN) to provide a prioritized set of cost-effective interventions (“best buys”) that are high-impact and feasible for implementation even in resource-constrained settings [10]. WHO PEN is a worthwhile approach for having managed NCDs, having progressed from its initial rollout in pilot sites of selected countries [2, 11] and showing promising results to becoming standard practice in countries such as Bhutan and Myanmar. For instance, in July 2017, the Prime Minister of Timor-Leste formally incorporated the PEN package into primary healthcare [12]. There is concern, however, about ensuring that the implementation of PEN interventions in matched with improving primary care facilities’ capacity to deliver these services since previous studies have shown large gaps between their capacity and WHO PEN standards [13–16]. As countries vary in terms of health system structure, economic development, and policies for addressing NCDs, there is a need to understand the gaps between the capacity of PHC facilities and delivering essential services to people with CMDs, especially grassroots facilities in LMICs.

Previous studies have investigated the capacity of primary healthcare facilities in delivering healthcare services for NCDs prevention; however, most have only analyzed internal facility capacity through cross-sectional surveys [13, 17, 18]. For example, one study conducted a cross-sectional survey of 90 PHC facilities in eight LMICs to measure the availability of factors such as human resources, equipment, and medicines [13]. While the major resource deficits were examined, the underlying reasons and related internal and external factors were not. Many commentary pieces have recommended that improving primary healthcare capacity in CMDs prevention and control needs political commitment and resource input, strong integration within the health system, connected care with other services, and awareness and acceptance from patients [19–21]. Therefore, we need to take a more comprehensive view to understand the capacity of primary healthcare facilities, especially with respect to interaction with patients and implementation of policies. In addition, we need to adopt mixed-methods to go beyond identifying key deficits and further understand the internal and external factors which influence capacity building and service delivery.

Another research gap is that most studies have focused on analyzing PHC facilities within specific healthcare systems [22, 23], while only a few have examined the common challenges and strategies that may be feasible regionally or across countries [13]. For example, grassroots PHC facilities may face difficulties in translating political commitment and national strategies into practice and have limited resources to invest in healthcare services. Thus, there is a need for cross-country studies to highlight the common challenges faced by grassroots PHC facilities and those strategies which may best facilitate PHC strengthening. By providing such a cross-country comparison, this study will make evidence-based recommendations for strategies which can be feasibly and broadly adopted across resource-limited settings.

In light of these research gaps, we will conduct the present study in resource-limited areas in four countries: China, Nepal, Vietnam, and Kenya.

### Study design

#### Overview

**Fig. 1:**
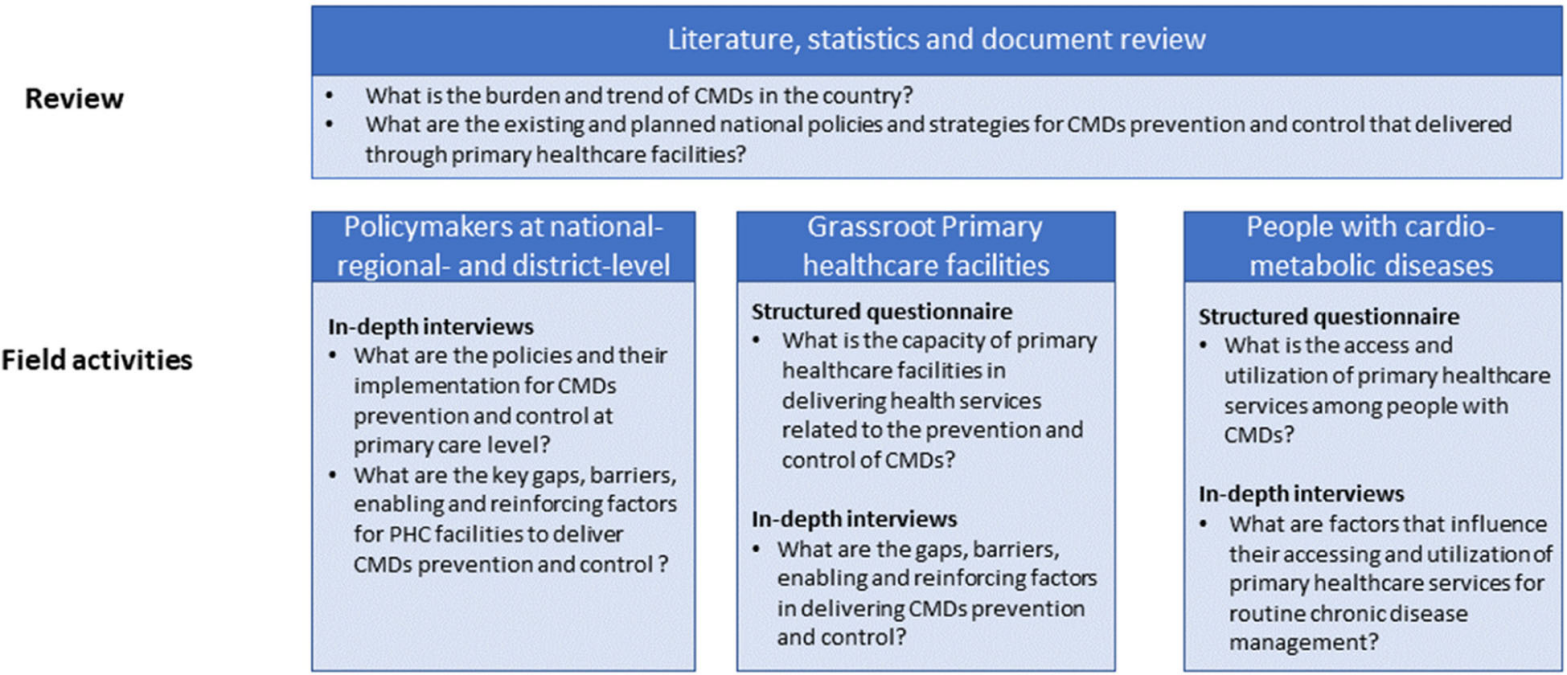
Overview of research activities and addressed research questions

For this study, we will utilize multiple research methods, including literature reviews, quantitative surveys, and qualitative interviews to collect data. Specifically, the study will:

1. Review literature, statistics, and documents to synthesize existing information on the burden and trends of CMDs as well as national policies for CMDs prevention and control, with a focus on PHC services in resource-limited settings;
2. Conduct facility assessment surveys to evaluate the capacity of PHC facilities and to perform in-depth interviews with leaders of PHC facilities to identify gaps, barriers, and enabling and reinforcing factors in delivering healthcare services related to CMDs prevention and control;
3. Perform cross-sectional surveys and in-depth interviews with people with CMDs to gain a deeper understanding of factors that influence patients’ accessibility and utilization of services, and
4. Conduct in-depth interviews among policymakers at national, regional and district level to understand the implementation of policy strategies and to identify factors that related to PHC system strengthening and NCDs related program implementation. Figure 1 provides an overview of the study design, the linkage of research activities, and the specific research question to be addressed.

The project protocol is developed by the core research team and will be implemented and adapted by researchers in four countries (China, Nepal, Vietnam, and Kenya) based on the local contexts. Research activities will be conducted at the national, regional, and facility level. All first-hand data collection will be conducted by the research team in the country, considering the familiarity with local languages, the country context, and the feasibility in study implementation. Table 1 shows the scope of fieldwork activities planned in each country.

**Table 1:** Overview of the scope of fieldwork research activities

Level of stakeholders	Activities
National Level	• At least 1 individual in-depth interview of a key national level policymaker ^a^
Regional Level (2 regions for each country)	For each region: • 2 individual in-depth interviews of regional level policymakers ^a^ • 2 individual in-depth interviews of district-level policymakers ^a^
Primary health care facility level (10 PHC facilities in total for each country)	For each primary health care facility: • 1 Facility Assessment Questionnaire • 1 individual in-depth interview of PHC provider/facility leader ^b^ • 10 structured questionnaires among people with CMDs • 1–2 individual in-depth interview among people with CMDs

Note: ^a^Policymakers include people who hold a position within the district, regional, or national government or an institution that plays a key role in making or implementing policies that relate to the primary health care system or preventing NCDs

^b^The PHC provider or facility leader needs to be knowledgeable to provide reliable information on behalf of their facility with at least 1 year working experience in the facility, good understanding of the capacity of the facility and NCD services

### Study setting

We will conduct this study in four countries: China, Kenya, Nepal and Vietnam. These four countries are similar in that they are all confronting the increasing burden of CMDs [24–27] and have plans to strengthen their primary healthcare systems to combat the increasing challenges of CMDs to the healthcare system. They are distinctive from each other, however, in demographic structure and socioeconomic development (see Table 2). The comparison of research findings across these four countries has the potential to demonstrate common gaps and challenges. In addition, the selection of these countries is based on funder requirements and our research collaboration network, which ensure the feasibility and quality in implementing the study protocol.

**Table 2:** Basic demographic and economic information of four countries in the study

	Kenya	Vietnam	China	Nepal
Basic characteristics ^a^				
Geographic location	Sub-Saharan Africa	Southeast Asia	East Asia	South Asia
Income level	Lower middle	Lower middle	Upper middle	Low
GDP at market prices in 2017 (US dollar)	$79.26 billion	$223.78 billion	$12.24 trillion	$24.88 billion
Population, total in 2017	49.70 million	95.54 million	1.386 billion	29.30 billion
Life expectancy at birth, total in 2016 (year)	67.0	76.2	76.3	70.3
Poverty headcount ratio at national poverty lines in 2017 (% of population)	36.1% (in 2015)	9.8% (in 2016)	3.1% (in 2017)	25.2% (in 2010)
Human Capital Index	0.52	0.67	0.67	0.49
The burden of NCDs and CMDs				
Proportional mortality due to NCD (% of all deaths) ^b^	27%	77%	89%	66%
Premature mortality from NCDs between 30 and 70 years (%)	13%	17%	17%	22%
Proportional mortality due to Cardiovascular diseases (% of all deaths) ^b^	8%	31%	43%	30%
Proportional mortality due to diabetes (% of all deaths) ^b^	1%	4%	2%	4%
DALYs per 100,000 at all ages due to cardiovascular diseases in 2017 ^c^	1967.13	4393.08	6020.67	3859.18
DALYs per 100,000 at all ages due to diabetes in 2017 ^c^	630.73	1109.21	709.68	708.95

Notes: ^a^Data from World Bank Data accessed through https://data.worldbank.org/

^b^Data from the World Health Organization, Noncommunicable disease country profiles 2018, accessed through https://www.who.int/nmh/publications/ncd-profiles-2018/en/

^c^Global Burden of Disease Collaborative Network. Global Burden of Disease Study 2017 (GBD 2017) Results. Seattle, United States: Institute for Health Metrics and Evaluation (IHME), 2018. Available from http://ghdx.healthdata.org/gbd-results-tool

In each country, the local research team will identify two representative resource-limited regions to conduct the study. The two areas will be selected based on resource level (below national average development level), diversity in geographical location, and willingness to participate. Five PHC facilities within each region will be selected by local collaborators based on the following four principles: (1) First contact: the selected PHC facilities should be at the grassroots level as the first contact point between the health system and the public. (2) Representativeness: the ten PHC facilities could represent the PHC facilities in resource-limited settings in the country, as in rural areas and urban slums. (3) Feasibility: researchers should be able to travel between PHC facilities under the planned budget. (4) Willingness: PHC facilities are willing to participate in the study and are able to provide reliable information to researchers.

### Literature, statistic, and document review

The purpose of the literature, statistic, and document review is to integrate existing information within our selected countries on (1) the burden and trends of CMDs, and (2) existing and planned national policies and strategies for CMDs prevention and control, focusing on PHC services in resource-poor settings. This information will provide a big picture overview of the challenges and existing strategies in each country and guide us to identify the gaps between the current PHC facility capacity and local needs. The review scope will be limited to national and regional representative information only published after the year 2000. International and national databases will be searched to identify the following types of studies and documents: (1) peer-reviewed literature from PubMed, Medline, Cochrane and EMBASE (peer-reviewed literature in local languages will also be reviewed if the local database exists and is accessible); (2) national/regional reports and grey literature; and (3) national-level statistics from international organizations or institutions such as the World Bank, WHO, Global Burden of Diseases, etc. Additional file 1 showed the details of the searching strategy for each aim. We will extract data to synthesize on the following information: (1) the disease burden of CMDs in the country; (2) policies related to primary healthcare strengthening or NCDs prevention and management; and (3) national or regional programs delivered through PHC facilities for promoting the prevention and management of CMDs.

### Field activity 1: primary health care facility assessment

We will use a structured questionnaire and conduct in-depth interviews to assess the capacity of PHC facilities in delivering health services related to the prevention and control of CMDs, as well as identify related gaps, barriers, and enabling and reinforcing factors.

#### Participant selection

A facility assessment questionnaire will be administered at each selected PHC facility by the local research team in each country. A knowledgeable PHC health care professional will participate in the study to provide reliable information on behalf of their facility. Participants are eligible if they have been working in the facility for no less than 1 year, have a good understanding of the capacity of the facility and CMD related services, and are willing to participate in the study. We will first contact the leader of the facility as the potential participant. If the leader of the facility does not fulfill the criteria, the leader will recommend an eligible person within the facility to participate in the study.

#### Instruments development

The facility assessment questionnaire is developed on the basis of the “WHO sample questionnaire for rapid assessment of capacity in primary care facilities” from the WHO PEN package [10], with revision focusing on CMDs. The questionnaire has five sections: “Equipment & service availability,” “Medicine,” “Medical record system,” “Service utilization & referral,” and “Financing & human resources.” In addition to the structured questionnaire, the local research team will also conduct semi-structured in-depth interviews among health care professionals. The interview guide consists of nine questions, covering the routine management of CMDs, secondary prevention of CMDs, and the “six building blocks of PHC” (health service delivery, health workforce, health information systems, access to essential medicines, health systems financing, and leadership and governance) [28]. Additional file 2 shows the facility assessment survey and the interview guide.

### Field activity 2: patients’ surveys and interviews

Structured questionnaires and in-depth interviews will be conducted to understand the utilization of PHC services among people with CMDs for their routine chronic disease management and to examine the obstacles preventing them from accessing such care in a timely manner.

#### Participants selection and sample size

We will apply a stratified convenience sampling approach to recruit participants. Ten CMD patients from each PHC facility (100 in total for each country) will be selected to participate in the structured questionnaire. Eligible participants are adults aged more than 18 years old, who have been diagnosed with heart disease, stroke, hypertension, and diabetes, and are willing to participate and give their consent. To achieve a more representative cohort, the local data collection team will take the following selection criteria into consideration if there are more than ten eligible participants from each facility: five males and five females; at least two participants less than 45 years old; people who have been diagnosed by a health care professional as having heart diseases and stroke will be recruited first; and at least two patients who have not obtained care from PHC facilities in the past year. The recruitment of participants will mainly rely on the staffs from community-based organizations or providers in the PHC facilities. We will conduct informed consent and screening for participant eligibility before administering the survey.

Data collectors will select survey participants to take part in the in-depth interviews if participants are able to communicate well and are willing to share more thoughts. The number of participants involved in the in-depth interview will be decided based on a saturation point of obtaining a comprehensive understanding without new information acquired. In general around ten participants from each country are expected.

#### Instrument design

The questionnaire for the patient survey is developed based on previous projects [29, 30]. The questionnaire includes five parts: demographic information and disease history, lifestyle risk factors, routine management, access to essential medicines, and satisfaction towards primary healthcare facilities. The in-depth interview guide aims to explore the gaps, barriers, enabling and reinforcing factors in utilizing the CMDs management and prevention services. It includes four open-ended questions on current routine management of CMDs, needs of CMD prevention and treatment, and comments on PHC services. Additional file 3 shows the patients’ survey and the interview guide.

### Field activity 3: policymaker interviews

In addition to the interviews of patients and health care professionals, we will conduct in-depth interviews among policymakers at the district, regional, and national level. The aim of these policymaker interviews is to identify the gaps, barriers, and enabling and reinforcing factors for policy and strategy implementation for strengthening primary healthcare.

#### Participants selection and sample size

Participants will be identified based on the support from the local collaborators and communities. Participants are eligible if they hold a position within the district, regional, or national government or an institution that plays a key role in making or implementing policies that relate to the PHC system or preventing NCDs.

#### Instruments design

The in-depth interviews will be conducted by trained researchers who speak the local languages and understand the local health system. The interview guide consists of open-ended questions covering the aspects of existing policies and action plans related to the prevention and control of NCDs, governance and leadership, factors related to the building blocks of healthcare system such as health financing, human resources, health service delivery, and health information systems that may facilitate the strengthening of the PHC system [28]. Additional file 4 shows the interview guide.

### Data collection

Trained researchers will conduct the structured facility assessment and patient questionnaires through face-to-face interviews. If the researchers have language difficulties, a translator with a good understanding of the protocol will be present with the researchers to assist in data collection. The survey will be collected using electronic data collection forms via tablet computers. If the local situation cannot fulfill the basic requirement of electronic data collection, we will adopt a pencil-and-paper approach and the local research team will double-enter the data.

Trained researchers who have experience in conducting qualitative research will conduct the In-depth interviews. Interviews will be conducted in the local language, audio recorded, and then transcribed, verbatim, in local languages for further analysis.

### Quality control

Pilot tests of instruments will be conducted in each country before the data collection to ensure feasibility under the local context. The instruments will be translated from English to the local language and back translated to ensure consistency across countries. Protocol training and technical training among data collectors will also be organized in each country before data collection.

### Data analysis and management

Descriptive analyses will be carried out using STATA statistical software (Version 15, College Station, TX, StataCorp LLC.). The analysis will be performed for each country. Mean and standard deviation will be reported for continuous variables with normal distribution; median and range will be reported if the data is skewed. Frequencies and proportions will be reported for categorical variables. For patients’ data, correlation analysis may be performed to explore the associated factors related to the utilization of PHC services on CMDs management.

For data collected through in-depth interviews, researchers will analyze the transcript in the local language by following a thematic analysis approach [31, 32]. Researchers will first read through all transcripts and then develop themes to capture the important features of the data. Codes will be generated for each transcript line by line and software may be used to facilitate the coding process based on local researchers’ capacity and preference. By categorizing the qualitative data into concepts and themes, we will report the key findings on the gaps and barriers of current PHC services on CMD management and prevention.

## Discussion

Strengthening PHC is one of the key strategies to combat the increasing burden of CMDs. With the epidemiological transition occurring in most LMICs, attention has begun to shift to the prevention and control of NCDs, including CMDs [2]. Such a shift poses considerable challenges to the health care system as most LMICs were not prepared for such change [33]. Health care systems have to consider future needs with an integrated approach that could combat the coexistence of persisting infectious diseases and emerging NCDs [20, 21, 34]. As the service-fronting element of the PHC approach, PHC facilities are vital in providing the linkage between the community and the health care system and translating government commitments and public health actions into community-based services [20]. Thus, the contribution of our study is to describe the trends of CMDs’ epidemic in within the selected countries, synthesize existing policies and strategies that promote PHC services on prevention and control of CMD, identify gaps in PHC capacity for implementing existing policy and strategies, and analyze the potential internal and external facilitators and barriers that may push forward the strengthening of PHC.

In this study, we will examine PHC facilities from multiple perspectives by using mixed-methods and involving various stakeholders, including patients, health care professionals, and policymakers. The resulting information on disease burden from the literature, statistical, and document review will provide an overview of the epidemic of CMDs in each country with respect to current trends and scope of the challenges. The review on existing policies and programs will provide researchers and stakeholders with an overview of the policy environment and the role of PHC facility within the whole healthcare system. The patient data will focus on factors related to service delivery, with the objective to cast into sharp relief the gaps between available services and the needs of patients. Compared with previous studies, which have solely assessed PHC facilities, our approach is able to provide a more comprehensive view on the current capacity of the grassroots primary health facilities in delivering services for people with CMDs and related internal and external factors. By understanding how grassroots PHC facilities interact with patients and policies, we will be able to identify potential interventions that are accessible, meet the needs of people with CMDs, can be feasibly delivered by grassroots PHC facilities in resource-limited settings, and may be sustainably supported by governments.

The focus of LMICs in this study has the potential to inform global efforts in promoting NCDs prevention and control in LMICs. Despite the growing evidence supporting the benefits of PHC-focused health systems in recent decades, there are still gaps in understanding the implementation of a primary care approach for NCD prevention and control in LMICs [35, 36]. On one hand, most LMICs are encountering a more complex situation with regard to the co-existence of communicable and NCDs. On the other hand, fragile health care systems in many LMICs are not adequately prepared for implementing the “best-buy” interventions [36, 37] that have been evaluated in high-income settings. This study has the potential to add new evidence and enlighten LMICs in ways to promote PHC in a horizontal approach with a specific vertical focus on CMDs.

This study is further unique in that it focuses on resource-limited settings in LMICs. The four countries – China, Nepal, Vietnam, and Kenya – share various similarities, notably an increasing burden of CMDs and related risk factors [24–27], and the inequity of health care resources and outcomes within the country [25, 38–40]. They are also distinct, however, with respect to population demographics, healthcare system structure, economic development, and so on. Difficulties in the prevention and control of CMDs may manifest differently across the four countries, but by highlighting the common challenges we can identify potential mitigating strategies in settings where the health workforce, financial input, and medical resources are below each country’s average level. In light of such evidence, we will identify potential solutions for implementing the best-buy interventions in resource-limited settings, which may lead to effective changes in risk factors and health outcomes among more vulnerable populations.

The study is a collaborative effort made by researchers from the four study countries and the results will be delivered to both the international audience and in-country stakeholders. High involvement of local researchers, who are experienced at working effectively within their respective countries, will ensure the effective implementation of the study with full consideration of local context and active involvement of key stakeholders. Local researchers will also be the key persons to deliver the study results to the country stakeholders to expand the study impact. We plan to deliver our study findings as policy briefs to attract a broader international audience. The specific country results will be published as peer-reviewed literature as well as a country-report to the policymakers in the country. Some in-country workshops on study findings will be organized, if feasible, to demonstrate study findings to PHC facilities leaders and discuss the potential action plans for improvement. With these dissemination plans, this project will not only provide evidence to the academic world but also has the potential to bring real-world impact to the local facilities, regions, or broadly.

After forty years since the declaration of Alma Ata [41], which characterized the role of PHC for the first time, researchers and policy makers are still holding our promise in promoting health for all through a PHC approach irrespective of diseases and settings [19, 20, 42]. With the WHO and LMIC governments increasingly committed to equity and universal health coverage, a focus on resource-limited settings in LMICs will bring new opportunities in generating feasible and effective strategies for combating the increasing burden of CMDs, especially among the most vulnerable populations.

## Supplementary information

**Additional file 1.** Literature, statistic and document review.

**Additional file 2.** Facility Assessment Questionnaire.

Additional file 3: Patient Questionnaire.

Additional file 4: Policy Maker interview guide.

## Data Availability

The datasets generated and/or analyzed during the current study are not publicly available but are available from the corresponding author on reasonable request.

## Abbreviations

CMDs: Cardiometabolic Diseases
LMICs: low- and middle-income countries
NCDs: Non-communicable Diseases
PHC: Primary Health Care
WHO PEN: World Health Organization “Package of Essential NCD interventions for primary care in low-resource settings”
