# Supplementary material for: Feasibility assessment of invigorating grassrooTs primary healthcare for prevention and management of cardiometabolic diseases in resource-limited settings in China, Kenya, Nepal, Vietnam (the FAITH study): rationale and design
Source: Glob Health Res Policy. 2019 Nov 12;4:33. doi: 10.1186/s41256-019-0124-0 (PMC6849318; doi:10.1186/s41256-019-0124-0)
Supplement: Supplementary file 1 — Additional file 1. Literature, statistic and document review. [file 41256_2019_124_MOESM1_ESM.docx]

## Additional file 1. Literature, statistic and document review

## Review purpose

The purpose of conducting literature, statistic and document review is to gather and synthesize existing information on the burden and trend of cardio-metabolic diseases (CMDs) and existing and planned national policies and strategies for CMDs prevention and control in the country with a particular focus on primary health care (PHC) services in resource-poor settings.

## Scope of the review and key sources

Considering the limited budget and human resources, we will limit the review scope to national and regional representative information only published after the year of 2000. International and national database were interrogated to identify the following types of studies and documents: (1) peer-reviewed literature from four electronic literature databases: PubMed, Medline, Cochrane and EMBASE. Peer-reviewed literature in local language will also be reviewed if the local database exists and is accessible; (2) national or regional reports and grey literatures, such as national statistical report, policy documents, etc. (3) national-level statistics from international organizations or institutions: such as World Bank, World Health Organization, Global Burden of Diseases, etc.

## Searching strategies:

## *CMDs Burden and Related Risk Factors*

We will focus on the statistical data that measure the burden of CMDs and related risk factors. The peer-reviewed literatures were limited to four databases, including PubMed, Medline, Cochrane and EMBACE. We will also look at literatures in local language if there is a local database. In addition, data from the World Health Organization, Global Burden of Diseases and National Statistical database will also be the key sources of national level statistical data.

The search was conducted using the following key terms:

1. CMDs: including stroke (ischemic stroke and hemorrhagic stroke), heart diseases (myocardial ischemia, coronary heart diseases, etc.), hypertension (as a risk factor) and diabetes (as a risk factor);
2. CMDs related risk factors: raised blood pressure, raised blood glucose, tobacco use, salt intake;
3. CMDs related Indicators: Incidence, prevalence, mortality, disability adjusted life years (DALYs), costs.

### ***Primary health care and Strategy on CMDs prevention and management***

Information about the structure, accessibility, and quality of the primary health care services in each country will also be included in the review. The searching source will mainly be published peer-reviewed literature, national/regional documents or reports, and grey literature. Additionally, the policy reports and documents about the strategy on CMDs/NCDs prevention and management at the national or regional level will also be reviewed. Original studies that assessed the implementation of related policies will also be included. National databases of policy reports will be considered as key sources of the review if it is accessible.
