# Supplementary material for: Feasibility assessment of invigorating grassrooTs primary healthcare for prevention and management of cardiometabolic diseases in resource-limited settings in China, Kenya, Nepal, Vietnam (the FAITH study): rationale and design
Source: Glob Health Res Policy. 2019 Nov 12;4:33. doi: 10.1186/s41256-019-0124-0 (PMC6849318; doi:10.1186/s41256-019-0124-0)
Supplement: Supplementary file 2 — Additional file 2. Facility Assessment Questionnaire. [file 41256_2019_124_MOESM2_ESM.docx]

**Additional file 2-A: Facility Assessment Questionnaire**

**FAITH Facility Assessment Questionnaire**

| Country: ____­­­­_________  Interviewer: ________________  Date: _____­/_____ (DD/MM)  Facility name: _______________________________________  Facility setting type: _1_ Urban _2_ Peri-urban _3_Rural  GPS Info: ________________/___________________  [Latitude] [Longitude] |
| --- |

| 1. **General Information**   Respondent name：**___**­­­­**_________**  Respondent job title：**___­­­­_________**  Respondent phone number: office phone **___________________**; cellphone **___________________**  Does this facility meet the national standards? _1_Yes _2_No  This facility serves: **____________________/_____________________/_____________________/__________________**  [province/ municipality] [provincial municipality] [town/ district] [commune/ ward]  How many primary health care facilities serve this area? **__________________**facilities  How many population this facility serves? **_________** households **_________** people |
| --- |
| 1. **Equipment & Service Availability**   Availability of basic equipment for CVDs management (number of **functional** devices available)  *Note: fill in “0” if there is none (this note applies to all the following questions involved with numbers)   \| Equipment \| Weighing machines \| Measuring tape \| Stethoscope \| Glucometer \| ECG machines \| \| --- \| --- \| --- \| --- \| --- \| --- \| \| Number \| **________** \| **________** \| **________** \| **________** \| **________** \|   Availability of Blood Pressure Measuring Devices (BPMDs):   \| Type \| Number \| How often are BPMDs calibrated for accuracy? \| \| --- \| --- \| --- \| \| Mercury \| **________** \| _1_Once a year or more _2_Less than once a year _3_Never _4_Don’t know \| \| Aneroid \| **________** \| _1_Once a year or more _2_Less than once a year _3_Never _4_Don’t know \| \| Automatic (non-portable) \| **________** \| _1_Once a year or more _2_Less than once a year _3_Never _4_Don’t know \| \| Automatic (portable & wrist cuff) \| **________** \| _1_Once a year or more _2_Less than once a year _3_Never _4_Don’t know \| \| Automatic (portable & upper arm) \| **________** \| _1_Once a year or more _2_Less than once a year _3_Never _4_Don’t know \|   Availability of information/ communication devices (facility owned or personally owned):   \| Device \| Classification \| Availability \| \| --- \| --- \| --- \| \| Internet \| Cable \| _1_Yes, stable _2_Yes, but not stable _3_No \| \| Wifi \| _1_Yes, stable _2_Yes, but not stable _3_No \| \| Computer \| Desktop \| _1_Yes _2_No \| \| Laptop \| _1_Yes _2_No \| \| Pad/ Tablet \| \| _1_Yes _2_No;  If yes, they are mainly based on: _1_Android _2_IOS _3_Window _4_Don’t know \| \| Cellphone \| Basic phone \| _1_Yes _2_No \| \| Smart phone \| _1_Yes _2_No;  If yes, they are mainly based on: _1_Android _2_IOS _3_Window _4_Don’t know \|   Availability of medical procedures and laboratory tests:   \| Injection \| IV fluid \| Blood sugar test \| Blood cholesterol test \| \| --- \| --- \| --- \| --- \| \| _1_Yes _2_No \| _1_Yes _2_No \| Finger tip _1_Yes _2_No  Venipuncture _1_Yes _2_No \| Finger tip _1_Yes _2_No  Venipuncture _1_Yes _2_No \|   Availability of counselling & educational services (if yes, tick on the box):   \| Patient counselling for: \| _1_Hypertension self-management _2_Diabetes self-management  _3_Neither _4_Don’t know \| \| --- \| --- \| \| Patient counselling and education on: \| _1_ Smoking _2_ Diet _3_Alcohol _4_Physical activity  _5_ None _6_ Don’t know \| \| Counselling and education of family members on: \| _1_ Smoking _2_ Diet _3_Alcohol _4_Physical activity  _5_ None _6_ Don’t know \|     Is there educational material for CVDs? If yes, who provide those materials?  _1_ No _2_Yes, by central government _3_Yes, by local government _4_Yes, by CDC _5_Yes, by private company  _6_ Yes, by NGOs _7_ Yes, self-made _8_ Yes, other (specify: **___________**) _9_ Don’t know |
| 1. **Medicine**  \| Medicine \| Availability \| Prescription right \| \| --- \| --- \| --- \| \| Aspirin \| _1_Always _2_Sometimes _3_No \| _1_Yes _2_No \| \| Atenolol \| _1_Always _2_Sometimes _3_No \| _1_Yes _2_No \| \| Enalapril \| _1_Always _2_Sometimes _3_No \| _1_Yes _2_No \| \| Furosemide \| _1_Always _2_Sometimes _3_No \| _1_Yes _2_No \| \| Isosorbide dinitrate \| _1_Always _2_Sometimes _3_No \| _1_Yes _2_No \| \| Statins (lovastatin or simvastatin) \| _1_Always _2_Sometimes _3_No \| _1_Yes _2_No \| \| Calcium channel blockers  (nifedipine retard, amlodipine) \| _1_Always _2_Sometimes _3_No \| _1_Yes _2_No \| \| Glyceryl trinitrate \| _1_Always _2_Sometimes _3_No \| _1_Yes _2_No \| \| Heparin \| _1_Always _2_Sometimes _3_No \| _1_Yes _2_No \| |
| 1. **Medical record system**   Does the facility keep a record of patient visit?  _1_Yes, records kept for all visits  _2_Yes, records kept for certain types of visit; specify: **____________________**  _3_No records kept  How is record kept?  _1_Paper based files  _2_Electronic health record  _3_Other; specify: **__________________**  What information will be recorded (if yes, tick on the box):  _1_ Date _2_ Name _3_ Symptom _4_ Examination results (_5_ Blood Pressure) _6_ Diagnosis _7_ Prescription  _8_ Others; specify: **_______________________________________** |
| 1. **Service utilization & Referral**  \|  \| Last week \| Last month \| Source \| \| --- \| --- \| --- \| --- \| \| Patients \|  \|  \|  \| \| In-patients \| **__________** \| **__________** \| _1_Record _2_Estimation \| \| Out-patients \| **__________** \| **__________** \| _1_Record _2_Estimation \| \| Home visit \| **__________** \| **__________** \| _1_Record _2_Estimation \| \| CVD patients \|  \|  \|  \| \| In-patients \| **__________** \| **__________** \| _1_Record _2_Estimation \| \| Out-patients \| **__________** \| **__________** \| _1_Record _2_Estimation \| \| Home visit \| **__________** \| **__________** \| _1_Record _2_Estimation \| \| Patients by appointment \| **__________** \| **__________** \| _1_Record _2_Estimation \| \| Patient referrals \| **__________** \| **__________** \| _1_Record _2_Estimation \|   *Note: Patients who stay at the facility over night are in-patients.  Can you refer patients to another facility due to CVDs in the following situations?   \|  \| Referral \| Distance (in kilometer) \| Referral institute name \| \| --- \| --- \| --- \| --- \| \| Emergency \| _1_Yes _2_No \| If yes _________________ \| _______________________ \| \| Additional test \| _1_Yes _2_No \| If yes _________________ \| _______________________ \| \| Specialist consultation \| _1_Yes _2_No \| If yes _________________ \| _______________________ \|     What means of transport is most frequently used to transfer emergency patients at your facility (check only one)?  _1_Ambulance _2_ Commercial vehicle (e.g. taxi) _3_ Private vehicle _4_ Public transportation  _6_Motorbike _7_Bike _8_Other; specify: **___________________** |
| 1. **Financing**   Revenue composition:   \|  \| Percentage \| \| --- \| --- \| \| Government budget \| **________________%** \| \| Social health insurance \| **________________%** \| \| Commercial health insurance \| **________________%** \| \| Patient out-of-pocket payment \| **________________%** \| \| Others, specify**________________** \| **________________%** \|   This revenue composition is based on: _1_Record _2_Estimation |
| 1. **Human resources**  \|  \| Number present today \| Total Number \| Total training times on CVDs among all staffs  (in the past year) \| Total self-study time on CVDs among all staffs  (in the past year) \| \| --- \| --- \| --- \| --- \| --- \| \| Specialists \| **________** \| **________** \| **________**times \| **________**times \| \| Doctors with board certificate \| **________** \| **________** \| **________**times \| **________**times \| \| Doctors without board certificate \| **________** \| **________** \| **________**times \| **________**times \| \| Pharmacists \| **________** \| **________** \| **________**times \| **________**times \| \| Nurses \| **________** \| **________** \| **________**times \| **________**times \| \| Health workers with payment \| **________** \| **________** \| **________**times \| **________**times \| \| Health workers without payment \| **________** \| **________** \| **________**times \| **________**times \| \| Administrative staffs \| **________** \| **________** \| **________**times \| **________**times \|   *Note: 1. Training/ Self-study time between 2 to 4 hours accounts for 1 time.  2. Training/ Self-study time between 5 to 8 hours accounts for 2 times. |
| That’s end. Thanks for your participation! |

**Additional file 2-B: Healthcare provider interview guide**

**FAITH study health PHC provider interview guide**

**Aim:** To identify gaps, barriers, enabling and reinforcing factors in delivering the cardiometabolic disease management and control at primary healthcare level in low-resource limited settings.

Interview will last for about 45 minutes. It will be recorded if consent is provided. Participation is completely voluntary and the interviewee has a right to withdraw participation during or after the interview.

**Step 0:** Fill in the information below

Date: _____­/_____­/_____ (MM/DD/YY)

Facility name: ________________________________________________

Facility code: _______________________

Name of interviewer: ________________

Interviewee Information:

Name: ________________

Age: __________________________

Job title: ______________________________

The job is: _1_ Full-time job _2_. Part-time job

Education background: ____________________________________

Hong long has the interviewee been worked in this facility: _____________ years

**Step 1:** Read out relevant sections in Consent Form to interview participants

**Step 2:** Consenting participants to sign the Consent Form

**Step 3:** Casually chat with the participant to establish rapport

**Step 4:** Ask the questions below:

1. **CVD prevention**
   1. *Population approach*

*Please describe how does your facility participate in CVD-related population approaches (smoking cessation campaign, healthy diet campaign, alcohol abstinence campaign, exercising campaign)? Has your facility ever met any problem or difficulty? What are some internal and external enabling factors?*

*Probe: enabling factors could come from the facility itself (e.g. skilled & experienced specialist, payment incentive, passion among health workers…) or from the outside of the facility (e.g. national policy, government earmarked subsidy, NGO campaign…)*

- 1. *Opportunistic high risk screening & high-risk patient management*

*Please describe how does your facility provide CVD opportunistic high-risk screening and CVD high-risk patient management. Has your facility ever met any problem or difficulty? What are some internal and external enabling factors?*

1. **CVD management**
   1. *Regular follow-up*

*Please describe how does your facility follow up CVD patients? Has your facility ever met any problem or difficulty when following up CVD patients? What are some internal and external enabling factors that help you to follow up CVD patients?*

- 1. *Referral*

*In what situation will your facility refer CVD patients to another facility? Describe the referral process. Has your facility ever met any problem or difficulty when referring CVD patients? What are some internal and external enabling factors that help you to refer CVD patients?*

- 1. *Acute CVD*

*Please describe how does your facility manage acute CVD patients. Has your facility ever met any problem or difficulty when managing acute CVD patients? What are some enabling factors that help you to manage acute CVD patients?*

*If the interviewer think he/she is still not getting enough information, the interviewer should ask the following questions.*

1. **SIX BUILDING BLOCKS**
   1. *Health service delivery*

*What services about CVD are available at your facility (population based health promotion program/ early detection of people at high risks/ early treatment of high risk patients/ rehabilitative care)? Who are responsible for delivering each service (the number and mix of staff, and how they work together)?*

- 1. *Health workforce*

*Do you think you have enough workforce in your facility to manage CVD patients? How do you think about your work load every day?*

- 1. *Health information system*

*Are you satisfied with the medical recording system now? Especially when manage CVD patients.*

*If no – Why not? Any suggestions to improve?*

*If yes – What are the satisfactory factors?*

*What do you think about the feasibility of future implementation of M-health to help prevent and manage CVD patients? What are the possible barriers?*

- 1. *Access to essential medicines*

*Have you experienced shortage of medicine in your facility? Do you think the medical equipment in your facility is enough to provide primary healthcare of CVDs?*

- 1. *Health system financing*

*What is your opinion on financing related with CVD prevention and management? It could be government budget/ social health insurance/ commercial health insurance/ out-of-pocket payment.*

- 1. *Leadership and Governance*

*Is there any CVD (especially hypertension) management and prevention guideline available in your facility?*

*If yes – Who provide the guideline? Can you show us the protocol/ guideline?*

*If no – Do you think your facility need such protocol/ guideline?*

*Are you aware of any CVD related policy from the government? What is your opinion?*

*Which department is in charge of CVD management and prevention of your facility? Do you think they are doing a good job? Do you have any suggestions?*

1. **Others**

*Do you have anything else to share with us about CVD issues in your facility?*

**Step 5**: Thank the interviewee for their time and hand over small gift.
