# Supplementary material for: Feasibility assessment of invigorating grassrooTs primary healthcare for prevention and management of cardiometabolic diseases in resource-limited settings in China, Kenya, Nepal, Vietnam (the FAITH study): rationale and design
Source: Glob Health Res Policy. 2019 Nov 12;4:33. doi: 10.1186/s41256-019-0124-0 (PMC6849318; doi:10.1186/s41256-019-0124-0)
Supplement: Supplementary file 3 — Patient Questionnaire. [file 41256_2019_124_MOESM3_ESM.docx]

**Additional file 3-A: Patient Questionnaire**

**S1 Basic Information**

| **S1.0** | **General Information**  Data collection date: \| d \| d **\| \|** m \| m \| m **\| \|** y \| y \| y \| y \|  Country Code_ _ Site ID _ _  Patient ID _ _ _ _ Interviewer ID_ _ |
| --- | --- |
| **S1.1** | **Gender of the patient**  ☐ _1_ Male ☐ _2_ Female ☐ _99_ Cannot be determined |
| **S1.2** | **Year of birth of the patient**  19___ |
| **S1.3** | **In total, how many years have you spent at school and full-time study excluding pre-school?**  ___ Year |
| **S1.4** | **What is your marital status?**  ☐_1_ Never married/never lived together ☐_2_ Married/living together  ☐_3_ Divorced/Separated ☐_4_ Widowed |
| **S1.5** | **Which of the followings best describes your main work status over the past 12 months?**  ☐_1_Government employee ☐_2_ Non-government employee ☐_3_ Self-employee ☐_4_ Non-paid ☐_5_Student ☐_6_ Homemaker ☐_7_ Retired ☐_8_ Unemployed ☐_9_ Unknown employed☐_10_ Other (please specify)_____ |
| **S1.6** | **How do you rate your health status compared to others of your age?**  ☐_1_ Very good ☐_2_ Good ☐_3_ Same ☐_4_ Bad ☐_5_ Very bad  ☐_99_ Don’t know/refused |
| **S1.7** | **Do you have a mobile phone?**  ☐_1_ Yes, basic phone (go to S1.9) ☐_2_ Yes, smart phone (go to S1.9)  ☐_3_ No (go to S1.8) |
| **S1.8** | **Do your family have a mobile phone?**  ☐_1_ Yes, basic phone(go to S1.9) ☐_2_ Yes, smart phone (go to S1.9)  ☐_3_ No (go to S2.1) |
| **S1.9** | **On a daily basis, how many messages do you receive and send on the mobile phone that you can reach?**  ☐_1_ 0 ☐_2_ 1 ☐_3_ 2 ☐_4_ 3 ☐_5_ 4 or more |

**S2 Disease History**

| **1.Hypertension/High Blood pressure** | |
| --- | --- |
| **S2.1** | **Have you ever had your blood pressure measured by a medical professional?**  ☐_1_ Yes (go to S2.1A) ☐_2_ No(go to S2.2)  Date of the last blood pressure measurement YY/MM/DD |
| **S2.1A** | **Have you ever been told by a medical professional that you have raised blood pressure or hypertension?**  ☐_1_ Yes ☐_2_ No (go to S2.2) |
| **S2.1B** | **First diagnosed by**  ☐_1_ Medical professional in the primary healthcare facilities  ☐_2_ Medical professionals in the regional or higher level hospitals  ☐ Others______________ |
| **S2.1C** | **How many years have you had hypertension?** □□ Years |
| **2. Diabetes/High Blood Glucose** | |
| **S2.2** | **Have you ever had your blood sugar measured by a medical professional?**  ☐_1_ Yes (go to S2.2A) ☐_2_ No(go to S2.3)  Date of the last blood sugar measurement YY/MM/DD |
| **S2.2A** | **Have you ever been told by a medical professional that you have raised blood sugar or diabetes?**  ☐_1_ Yes ☐_2_ No (go to S2.3) |
| **S2.2B** | **First diagnosed by**  ☐_1_ Medical professional in the primary healthcare facilities  ☐_2_ Medical professionals in the regional or higher level hospitals  ☐_3_  Others______________ |
| **S2.2C** | **How many years have you had diabetes?** □□ Years |
| **3. Heart diseases** | |
| **S2.3** | **Have you ever had a heart attack or chest pain from heart disease (angina)?**  ☐_1_ Yes ☐_2_ No (go to S2.4) |
| **S2.3A** | **First diagnosed by**  ☐_1_ Medical professional in the primary healthcare facilities  ☐_2_ Medical professionals in the regional or higher level hospitals  ☐_3_ Traditional healer ☐_4_ Others______________ |
| **S2.3B** | **When was it first diagnosed** □□□□ year |
| **S2.3C** | **When was the latest event** □□□□ year |
| **4. Stroke** | |
| **S2.4** | **Have you ever had a stroke before?**  ☐_1_ Yes ☐_2_ No |
| **S2.4A** | **First diagnosed by**  ☐_1_ Medical professional in the primary healthcare facilities  ☐_2_ Medical professionals in the regional or higher level hospitals  ☐_3_ Traditional healer ☐_4_ Others______________ |
| **S2.4B** | **Type of stroke?** ☐_1_ Ischemic stroke ☐_2_ Hemorrhagic stroke ☐ _99_ Don’t know |
| **S2.4C** | **When was it first diagnosed □□□□ year** |
| **S2.4D** | **When was the last time that you had a stroke**  □□□□ year |

**If the participants answered NO to all of S2.1A, S2.2A, S2.3 and S2.4, then end questionnaire here as the participants do not satisfy our inclusion criteria.**

1. **Lifestyle Information**

| **A1** | **During the past month, on about how many days did you drink any alcoholic beverage?**  ☐_1_ 0 (go to A4) ☐ _ _ days |
| --- | --- |
| **A2** | **During the past month, on the days when you drank, about how many alcoholic drinks did you have, on average?**  ☐_1_ 0 (go to A4) ☐_2_ 1 ☐_3_ 2 ☐_4_ 3 ☐_5_ 4 ☐_6_ ≥5 |
| **A3** | **During the past month, considering all types of alcoholic beverages, how many times did you have ≥ 5drinks (males) or ≥ 4 drinks (females) on one occasion?**  ☐_1_ 0 ☐_2_ 1 ☐_3_ 2 ☐_4_ 3 ☐_5_ 4 ☐_6_ ≥5 |
| **A4** | **Have you ever smoked cigarettes in your entire life?**  ☐ _1_ Yes, more than 100 ☐ _2_ Yes, less than 100 ☐ _3_ No (Go to A7) |
| **A5** | **Do you currently smoke cigarettes every day, some days, or not at all?**  ☐ _1_ Everyday ☐ _2_ Somedays ☐ _3_ Not at all (Go to A7) |
| **A6** | **On average, when you smoke during the past month, about how many cigarettes did you smoke a day?**  ____cigarettes/day |
| **A7** | **In the past 3 months, on average, how many servings of fruit (fresh, frozen, or stewed) did you eat per day? Do not include fruit juice, canned fruit or dried fruit. Please select one answer only.**  ☐ _0_ I don’t eat fruit ☐ _3_ 2 servings per day  ☐ _1_ Less than 1 serving per day ☐ _4_ 3 servings per day  ☐ _2_ 1 servings per day ☐ _5_ 4 or more servings per day |
| **A8** | **In the past 3 months, on average, how many servings of vegetable (fresh and frozen) do you eat per day? Do not include vegetable juice. Please select one answer only.**  ☐ _0_ I don’t eat vegetable ☐ _3_ 2 servings per day  ☐ _1_ Less than 1 serving per day ☐ _4_ 3 servings per day  ☐ _2_ 1 servings per day ☐ _5_ 4 or more servings per day |
| **A9** | **During the last 7 days, on how many days did you do vigorous physical activates like heavy weight lifting, fast cycling, running, swimming, or basketball or soccer? Think only about those physical activities that you did for at least 10 minutes at a time.**  ___Days per week ☐ _99_ Don’t know/Refused |
| **A10** | **How much time did you usually spend doing vigorous physical activities on one of those days?**  ___Minutes per day ☐ _99_ Don’t know/Refused |
| **A11** | **During the last 7 days, how many days did you do moderate physical activities like speed walking, light weight lifting, dancing, or normal cycling? Think only about those physical activities that you did for at least 10 minutes at a time.**  ___Days per week ☐ _99_ Don’t know/Refused |
| **A12** | **How much time did you usually spend doing moderate physical activities on one of those days?**  ___Minutes per day ☐ _99_ Don’t know/Refused |
| **A13** | **During the last 7 days, on how many days did you walk for at least 10 minutes at a time?**  ___Days per week ☐ _99_ Don’t know/Refused |
| **A14** | **How much time did you usually spend walking on one of those days?**  ___Minutes per day ☐ _99_ Don’t know/Refused |
| **A15** | **During the last 7 days, how much time did you usually spend sitting on a week day?**  ___Hours ___Mins per weekday ☐ _99_ Don’t know/Refused |
| **A16** | **Did you see a medical professional for medical reasons within the last 3 months**  **☐1 Yes ☐2 No *(if No go to B1)*** |
| **A17** | **During the past 3 months, has a medical professional told you to?**   1. Quit using tobacco or don’t start ☐_1_ Yes ☐_2_ No 2. Reduce the use of alcohol/don’t start ☐_1_ Yes ☐_2_ No 3. Reduce salt in your diet ☐_1_ Yes ☐_2_ No 4. Reduce use of refined sugar in your diet ☐_1_ Yes ☐_2_ No 5. Eat at least five servings of fruit and/or vegetables each day ☐_1_ Yes ☐_2_ No 6. Reduce fat in your diet ☐_1_ Yes ☐_2_ No 7. Start or do more physical activity ☐_1_ Yes ☐_2_ No 8. Maintain a healthy body weight or lose weight ☐_1_ Yes ☐_2_ No |

1. **Access to Primary Healthcare facility**

| **B1** | **How long does it take you to go to the nearest primary health facility that you know of?**  ___Hours ___Minutes |
| --- | --- |
| **B2** | **How would you rate this travel time?**  ☐_1_ Very long ☐_2_ Long ☐_3_ Reasonable ☐_4_ Short ☐_5_ Very short  ☐_99_ Don’t know |
| **B3** | **How do you usually go to your chosen primary healthcare facility ?**  ☐_1_ Bus ☐_2_ Car (for example, own car or taxi) ☐_3_ Motorbike ☐_4_Bicycle ☐_5_ Walked  ☐_6_Other (please specify)……………………………………………….  ☐_7_ I have never been to a primary healthcare facility. *(if never skip to C1)* |
| **B4** | **How much will be the total cost of transportation for this visit? Include the cost (to and from) for the patient and any necessary accompanying individuals.**  ________ local currency ☐_99_ Don’t know/Refused |
| **B5** | **How would you rate the cost of transportation?**  ☐_1_ Very expensive ☐_2_ Somewhat expensive ☐_3_ Neither inexpensive nor expensive  ☐_4_ Somewhat inexpensive ☐_5_ Very inexpensive or free |
| **B6** | **How long did you wait between arriving at the facility and receiving medical attention?**  ___Hours ___Minutes |
| **B7** | **How would you rate this waiting time?**  ☐_1_ Very long ☐_2_ Long ☐_3_ Reasonable ☐_4_ Short ☐_5_ Very short  ☐_99_ Don’t know/Refused |

1. **Treatment and control of Cardiovascular diseases**

| **C1** | **In the past 12 months, on average, how many times have you sought healthcare for any cardiovascular disease (as mentioned in section S2 above ) at this primary healthcare facility?**  ☐_1_ Never (**go to C2A**) _____Times (**go to C2B**) ☐_99_ Don’t know/Refused |
| --- | --- |
| **C2A** | **Why did you NOT come to this facility for cardiovascular diseases treatment? (*tick all applicable*)**  ☐_1_ Unfriendly staff ☐_2_ Long waiting time ☐_3_Medicine unavailable ☐_4_Staff are unqualified  ☐_5_ Services are expensive ☐_6_ Dirty facility ☐_7_ Would have paid ☐_8_ No privacy ☐_9_ Was referred ☐_10_ Beds not available ☐_11_ Distance too far ☐_12_ Other (specify)_____(**go to C10**) |
| **C2B** | **You came to this facility to seek treatment for (select all that apply)**  ☐_1_ diabetes (high blood glucose) ☐_2_ hypertension (high blood pressure)  ☐_3_ heart diseases ☐_4_ stroke ☐_5_ others ☐_99_ Don’t know/Refused  (**if answer include 1,2,3,4 go to C3,otherwise go to C11**) |
| **C3** | **What procedure did you go through when you come to this facility during the most recent CVD related visit? (Select all that apply)**  ☐_1_ Testing blood pressure ☐_2_ Testing blood glucose ☐_3_ ECG ☐_4_ Consultation ☐_5_ Obtain medication ☐_6_ Others(specify)_____ |
| **C4** | **What was the total amount you paid for medically-related fees during the most recent CVD related visit? This includes formal and informal payments** for tests, procedures, medical consultation, user fee, registration, and any other medical services. It also includes the cost of any medical supplies you purchased or provided for your visit and medication costs (***Record 0 if all services were free)***  _________(local currency) |
| **C5** | **How much of the total costs is covered by your insurance**? ___%  ☐_99_ Don’t know/Refused |
| **C6** | **How would you rate the total amount you paid for medically-related fees during the most recent CVD related visit?**  ☐_1_ Very expensive ☐_2_Somewhat expensive ☐_3_Neither inexpensive nor expensive  ☐_4_Somewhat inexpensive ☐_5_Very inexpensive or free |
| **C7** | **Were you prescribed any new medication at this visit?**  ☐_1_ Yes ☐_2_ No (go to C11) |
| **C8** | **Did the staff tell you what the medication was for?**  ☐_1_ Yes ☐_2_ No |
| **C9** | **Were all the medications you needed available at this facility?**  ☐_1_ Yes *(if YES, skip to C11)* ☐_2_ No |
| **C10** | **Why were you unable to obtain the medicines from this facility during the most recent CVD related visit?**  ☐_1_ Too expensive ☐_2_ Medicine(s) not in stock ☐_3_ Referred elsewhere by the medical professional ☐_4_ Other (Specify:____) ☐_99_ Don’t know/Refused |
| **C11** | **Do you know where to go for treatment if you suffer from the following symptoms**  **(chest pain, heart attack, lightness or pain in the chest, neck, back, or arms, as well as fatigue, lightheadedness, abnormal heartbeat, and anxiety as a result of stroke or heart attack)?**  ☐_1_ Don’t know/Refused ☐_2_ Yes **(list names of the facilities below)**  **1.______ 2. _____ 3._______** |

**D Medication History**

| **D1** | **In the last one month, have you been on any medication for CVD conditions diagnosed?** ☐_1_ Yes **Condition (if yes) :________**  ☐_2_ No *(if no skip to E1)* |
| --- | --- |
| **D2** | **To your knowledge, how many of those medication is for the treatment of CVD?**  **___________ (if 0 go to E)** |
| **D3** | **Who prescribes the CVD medications for you?**  ☐_1_ by myself ☐_2_ Nurse/Medical Officer at primary care facilities ☐_3_ Health staff from hospitals ☐_4_ Others please specify____ |
| **D4** | **Where do you usually get your CVD medications? *(Select all that apply)***  ☐_1_ This facility ☐_2_ Pharmacy at the facility where this clinic is located ☐_3_ Another facility(specify name) ☐_4_ Pharmacy elsewhere ☐_99_ Don’t know/Refused |
| **D5** | **What was the total formal cost of medicines for the past month? (Record 0 if free)**  _________ (local currency) |
| **D6** | **How would you rate the cost of medicines in the past months?**  ☐_1_ Very expensive ☐_2_Somewhat expensive ☐_3_Neither inexpensive nor expensive  ☐_4_Somewhat inexpensive ☐_5_Very inexpensive or free |
| **D7** | **Do you have difficulties taking your medications as prescribed?**  ☐_1_ Yes ☐_2_ No |
| **D8** | **Do you have unanswered questions about your medications?**  ☐_1_ Yes ☐_2_ No |
| **D9** | **Do you ever forget to take medication?**  ☐_1_ Yes ☐_2_ No |
| **D10** | **What kind of difficulty did you have in obtaining your/their CVD medications?**  ☐_1_ Pharmacy or clinic closed ☐_2_ Medication unavailable or out of stock ☐_3_ Too expensive ☐_4_ Other (Specify_______) |

If participant selected option 1 (Never) in **C1**, then end survey here. Otherwise, continue to Section E.

**E Patient Satisfaction**

| **E1** | **Please indicate to what extent you agree or disagree with the statements below**   1. **During this visit, medical professionals treated me with courtesy and respect**   ☐_1_ Strongly Disagree ☐_2_ Disagree ☐_3_ Agree ☐_4_ Strongly Agree   1. **During this visit, medical professionals listened carefully to me**   ☐_1_ Strongly Disagree ☐_2_ Disagree ☐_3_ Agree ☐_4_ Strongly Agree   1. **During this visit, medical professionals explained things in a way I could understand**   ☐_1_ Strongly Disagree ☐_2_ Disagree ☐_3_ Agree ☐_4_ Strongly Agree   1. **The facility was clean**   ☐_1_ Strongly Disagree ☐_2_ Disagree ☐_3_ Agree ☐_4_ Strongly Agree   1. **The bathroom/latrines were clean (leave blank if not applicable)**   ☐_1_ Strongly Disagree ☐_2_ Disagree ☐_3_ Agree ☐_4_ Strongly Agree   1. **I had enough time to discuss my medical problem with the medical professionals**   ☐_1_ Strongly Disagree ☐_2_ Disagree ☐_3_ Agree ☐_4_ Strongly Agree |
| --- | --- |
| **E2** | **On a scale of 0-10 (0 being the worst facility, 10 being the best facility), how would you rate this health facility?**  ☐_1_ 1 ☐_2_ 2 ☐_3_ 3 ☐_4_ 4 ☐_5_ 5 ☐_6_ 6 ☐_7_ 7 ☐_8_8 ☐_9_ 9 ☐_10_ 10 |
| **E3** | **Would you recommend this facility to your friends and family?**  ☐_1_ Definitely no ☐_2_ Probably no ☐_3_ Probably yes ☐_4_ Definitely yes  **Transport reimbursement** ☐_1_ Yes ☐_2_ No |

**Additional file 3-B: Patients’ interview guide**

**FAITH Study Patients’ Interview Guide**

**Aim:** To explore cardiovascular (CVDs) patients’ routine management needs in primary healthcare setting (PHC), as well as the factors associated with the access and utilization patterns of PHC in meeting such needs.

Our discussion may last up to 45 minutes based on the four questions we will ask. You may talk as much as you prefer. Our discussion will be recorded if consent is provided. Confidentiality will be maintained unless the informant wishes his/her name to be mentioned in published reports resulting from the study. Participation is completely voluntary and the interviewee has a right to withdraw participation during or after the interview. Where needed a translator will be present to assist the researcher.

**Step 1**: Read out relevant sections in Consent Form to interview participants

**Step 2**: Consenting participants to sign the Consent Form

**Step 3**: Casually chat with the participant to establish rapport

**Step 4**: Ask the questions below:

*Question 1: How are you managing your conditions nowadays? What is your preferred routine management method for cardiovascular diseases?*

*Question 2: Do you think you are getting all the care you need to manage your CVD on a daily basis? If not, what is preventing from receiving treatment?*

*Question 3: If you visit health centers to receive care, what are some of the goods things you enjoy about those facilities? Any things that you dislike about them? What will make you want to get your routine CVD care from this kind of facilities?*

*Question 4: Do you know where you can seek emergency CVD care should you have a heart attack or stroke? If you have had a heart attack or stroke before, how did you manage it and where did you seek help?*

*Question 5: Is there anything that you think I should know about your (diabetes/hypertension etc)*

**Step 5**: Thank the interviewee for their time.
